# Oestrogen increases the activity of oestrogen receptor negative breast cancer stem cells through paracrine EGFR and Notch signalling

**DOI:** 10.1186/bcr3396

**Published:** 2013-03-08

**Authors:** Hannah Harrison, Bruno M Simões, Lynsey Rogerson, Sacha J Howell, Göran Landberg, Robert B Clarke

**Affiliations:** 1 Molecular Pathology Group, Breakthrough Breast Cancer Research Unit, Institute of Cancer Sciences, University of Manchester, Paterson Institute for Cancer Research, Manchester, M20 4BX, UK; 2 Breast Biology Group, Institute of Cancer Sciences, University of Manchester, Paterson Institute for Cancer Research, Manchester, M20 4BX, UK; 3 Department of Medical Oncology, The University of Manchester, The Christie NHS Foundation Trust, Manchester, M20 4BX, UK; 4 Sahlgrenska Cancer Center, Institute of Biomedicine, 405 30 Göteborg, Sweden

## Abstract

**Introduction:**

Although oestrogen is essential for the development of the normal breast, adult mammary stem cells are known to be oestrogen receptor alpha (ER) negative and rely on paracrine signals in the mammary epithelium for mediation of developmental cues. However, little is known about how systemic oestrogen regulates breast cancer stem cell (CSC) activity.

**Methods:**

Here, we tested the effects of oestrogen on CSC activity *in vitro *and *in vivo *and investigated which paracrine signalling pathways locally mediate oestrogen effects.

**Results:**

CSC-enriched populations (ESA^+^CD44^+^CD24^low^) sorted from ER positive patient derived and established cell lines have low or absent ER expression. However, oestrogen stimulated CSC activity demonstrated by increased mammosphere and holoclone formation *in vitro *and tumour formation *in vivo*. This effect was abrogated by the anti-oestrogen tamoxifen or ER siRNA. These data suggest that the oestrogen response is mediated through paracrine signalling from non-CSCs to CSCs. We have, therefore, investigated both epidermal growth factor (EGF) and Notch receptor signals downstream of oestrogen. We demonstrate that gefitinib (epidermal growth factor receptor (EGFR) inhibitor) and gamma secretase inhibitors (Notch inhibitor) block oestrogen-induced CSC activity *in vitro *and *in vivo *but GSIs more efficiently reduce CSC frequency.

**Conclusions:**

These data establish that EGF and Notch receptor signalling pathways operate downstream of oestrogen in the regulation of ER negative CSCs.

## Introduction

Normal mammary stem cells (MSC) are responsible for the generation of adult mammary tissue and the distinct cell types within it as well as the extensive remodelling and enlargement of the gland during multiple cycles of pregnancy [1,2]. Mammary development is controlled by a variety of hormones, including oestrogen without which development cannot occur [3]. Isolation of MSC using cell sorting techniques has allowed extensive studies of this cell sub-population and it has been shown that these cells lack oestrogen receptor alpha (ER) [4,5]. In order to respond to systemic hormone signalling, these cells must, therefore, rely on local mediation of the signals by ER positive cells. There is good evidence that the epidermal growth factor receptor (EGFR) pathway, via binding of the amphiregulin ligand, is responsible for paracrine signalling that induces epithelial proliferation during ductal elongation of the mammary tree, but it is unknown whether this signal affects stem cells [6].

The development and progression of breast tumours has been proposed to be driven by breast cancer stem cells (CSC) identified by the cell surface phenotype ESA^+^CD44^+^CD24^low ^or aldehyde dehydrogenase (ALDH1) activity [7,8]. CSCs generate tumour heterogeneity and are able to reinitiate tumours in transplantation experiments [7]. CSCs are thought to be responsible for tumour recurrence as they have been shown to be inherently resistant to therapies, such as chemotherapy [9], radiotherapy [10] and endocrine treatment [11,12].

There have been conflicting reports about the effects of oestrogen on breast CSCs with evidence reported that oestrogen can increase or decrease CSC number in breast cancer cell lines [13,14]. We predicted that these very different effects were due to the duration of hormone deprivation [12] compared to growth in standard conditions. In the current study, where oestrogen treatment is initiated after hormone withdrawal, oestrogen increases CSC activity and frequency measured both *in vitro *and *in vivo*. This is likely to be through paracrine regulation since breast CSCs are mainly ER negative. Here we establish that the EGF and Notch receptor signalling pathways are strong candidates as paracrine mediators of oestrogen effects on CSC activity.

## Materials and methods

### Patient samples

Pleural effusion samples (*n *= 3, see Additional file 1, Table S1) were collected from patients with metastatic breast cancer during standard therapeutic drainage procedures, with fully informed consent (ethical approval was granted by the Central Office for Research Ethics Committee, study #05/Q1403/159). Following collection of metastatic fluid, cells were pelleted by centrifugation at 800 g. Pellets were resuspended in PBS and blood cells were removed by centrifugation of the cell suspension through 0.5 volumes of Lymphoprep solution (Axis Shield, Dundee, UK) at 600 g. Cells were cultured in DMEM:F12/20% FCS/0.1% non-essential amino acid solution/2.5 mM L-glutamine/PenStrep (Invitrogen, Paisley, UK).

### Cell lines

Cell lines were purchased from the LGC Standards (Middlesex, UK); MCF7 (HTB-22), T47D (HTB-133), BT474 (HTB-20), MDA-MB-231 (HTB-26), authenticated by multiplex PCR assay using the AmpF/STR system (Life Technologies, Paisley, UK) and verified as mycoplasma free.

Monolayers of MCF7, T47D and BT474 were grown adherently in DMEM complete medium (DMEM/10% foetal calf serum/2 mM L-glutamine/PenStrep) and MDA-MB-231 were cultured as monolayer in RPMI complete medium (RPMI/10% FCS/1% Sodium pyruvate/2 mM L-glutamine/PenStrep). Cells were maintained in a humidified incubator at 37°C at an atmospheric pressure of 5% (v/v) carbon dioxide/air. Cells were passaged at 80% confluence with a sub-cultivation ratio of 1:4. Cell lines were not cultured beyond 20 generations.

### Clonogenic culture

Cells were plated at 50 cells/cm^2 ^in adherent conditions for 10 days (MCF7, T47D) or 6 days (MDA-MB-231). Colonies were fixed and stained with 1% crystal violet/70% ethanol and were classified by light microscopy. Colonies that had undergone five or more divisions, that is, containing 32 or more cells, were counted [15].

### Mammosphere culture

A single cell suspension was prepared using enzymatic (0.125% Trypsin-EDTA (Worthington Biochemical Corporation, Lakewood, NJ, USA)), and manual disaggregation (25 gauge needle). A total of 500 cells/cm^2 ^were plated in non-adherent conditions in mammosphere medium (DMEM-F12/B27/20 ng/ml EGF/PenStrep) for five days. Mammospheres (MS) greater than 50 μm were counted.

### Flow cytometry

Samples were resuspended at ≤ 1 × 10^6 ^in 100 μl sorting buffer (1% BSA/PBS) and incubated with 10 μl of primary pre-conjugated antibody for 10 minutes at 4°C. Following incubation the cells were washed with 1 ml PBS and centrifuged at 800 g for two minutes. Antibodies include ESA-FITC (Dako, Cambridge, UK, BerEP4, F0860), CD44-APC (BD Pharmingen, Oxford, UK, 559942) and CD24-PE (Beckman Coulter, High Wycombe, UK, IMI1428U)

For analysis, cells were resuspended in 500 μl of sorting buffer and passed through a 40 μm sieve. Fluorescence was measured using the Becton Dickinson FACS Calibur and analysed using WinMDI 2.8 software (The Scripps Institute, La Jolla, CA, USA).

For sorting, cells were resuspended in 500 μl of Hank's buffered saline solution (HBSS, Sigma, Cambridge, UK) and passed through a 40 μm sieve. Cells were sorted, with HBSS as a sheath fluid, at 16 PSI using the Becton Dickinson FACS ARIA (Oxford, UK).

### Quantitative RT-PCR

RNA was extracted using the RNAeasy kit (Qiagen, Manchester, UK) according to manufacturer's instructions and RNA was quantified using the Nanodrop spectrophotometer (Thermo Fisher Scientific, Basingstoke, UK). cDNA was produced using the Taqman reverse transcription kit (Life Technologies). qRT-PCR was performed using Sybrgreen (Bioline, London, UK) and was analysed on the 7900 PCR machine (Life Technologies) using custom PCR array plates (SA Biosciences, West Sussex, UK).

### Western blotting

Protein was separated on an SDS-polyacrylamide gel and transferred to Hybond-C Extra nitrocellulose membrane (GE Healthcare, Buckinghamshire, UK). Primary antibodies included: SP1-ER (RM-9101-SO, Thermo Fisher Scientific, Basingstoke, UK), Cleaved N1-ICD (100-401-407, Rockland, Gilbertsville, USA), Delta1 (Santa Cruz Biotechnology, Santa Cruz, CA, USA, sc-12530), Delta4 (Abcam, Cambridge, UK, ab7280), Jagged1 (Santa Cruz Biotechnology, sc-6011), Jagged2 (Santa Cruz Biotechnology, sc-08157), Actin (Santa Cruz Biotechnology, sc-1616), ERK (Abcam, ab2430) and ERK phospho-Y992 (Abcam, ab81440). Densitometry was performed using ImageJ software (NIH, Bethesda, USA) which is freely available [16]. Mean band intensity was measured and fold change from actin control was calculated.

### Oestrogen treatment

Cells were plated in a monolayer at 1 × 10^4^/cm^2 ^in complete medium for 24 hours. At 24 hour intervals the medium was changed to low serum medium with decreasing concentrations of charcoal stripped serum, from 10% to 1%. Charcoal stripped serum was prepared by mixing foetal calf serum with dextran coated charcoal and heating for 30 minutes at 55°C. Serum was then centrifuged at 1,000 g for 15 minutes to remove charcoal. Finally the serum was passed through a 0.22 μm sieve.

Cells were then cultured for 48 hours with 0 to 1 μM 17β-estradiol to identify the best concentration. A total of 1 nM was selected as the lowest concentration which gave significant changes (see Additional file 2, Figure S1) and was used with or without 1 μM 4OH-tamoxifen.

### siRNA

A SMARTpool of siRNA to ER (Thermo Scientific Dharmacon, ON-TARGET plus Human ESR1 (Fermentas GBMH, St Leon-Rot, Germany) was used to transfect cells using Lipofectamine 2000 (Invitrogen, Paisley, UK) according to the manufacturer's instructions in serum free medium. After six hours medium was changed to complete medium and cells were cultured as described above.

### Inhibition of paracrine signalling

Inhibitors of Notch (10 μM DAPT) and EGFR (1 μM gefitinib) signalling were added to oestrogen activated cell cultures (1 nM 17 β-estradiol) at Day 1 of treatment.

### In vivo limiting dilution

Cells were treated as detailed above in CSS medium ± 17β-estradiol ± inhibitors for 48 hours. Cells were collected and resuspended at the desired dilution (10, 100 and 1,000 cells) in 50% Matrigel (BD Biosciences, Oxford, UK)/MS medium before sub-cutaneous injection into NOD SCID IL2gammaR knock out (NSG) mice. Slow release oestrogen pellets were implanted sub-cutaneously into mice two days before cell injection (0.72 mg, Innovative Research of America (Sarasota, USA). Mice were assessed for tumour presence twice weekly.

### Xenograft embedding and immunohistochemistry

Tumours were formalin fixed and paraffin embedded. Antigen retrieval was performed at 98°C at pH 9. Slides were blocked with hydrogen peroxide and casein before incubation with the primary antibody (PanCytokeratin - #70622, DAKO, Cambridge, UK). Slides were then incubated with Envision secondary followed by DAB (DAKO).

### Statistical methods

Throughout the paper data are represented as mean ± SEM taken over a minimum of three independent experiments, unless otherwise stated. Statistical significance was measured using parametric testing, assuming equal variance, in the majority of experiments with standard t-tests for two-paired samples used to assess difference between test and control samples.

Analysis of variance was performed to assess changes in *in vivo *tumour growth rate. To calculate CSC frequency, the L-Calc software (The Walter and Eliza Hall Institute of Medical Research, Parkville, USA) was used which is freely available [17].

## Results

### Oestrogen stimulation increases the breast cancer stem cell-like population

**Figure 1:**
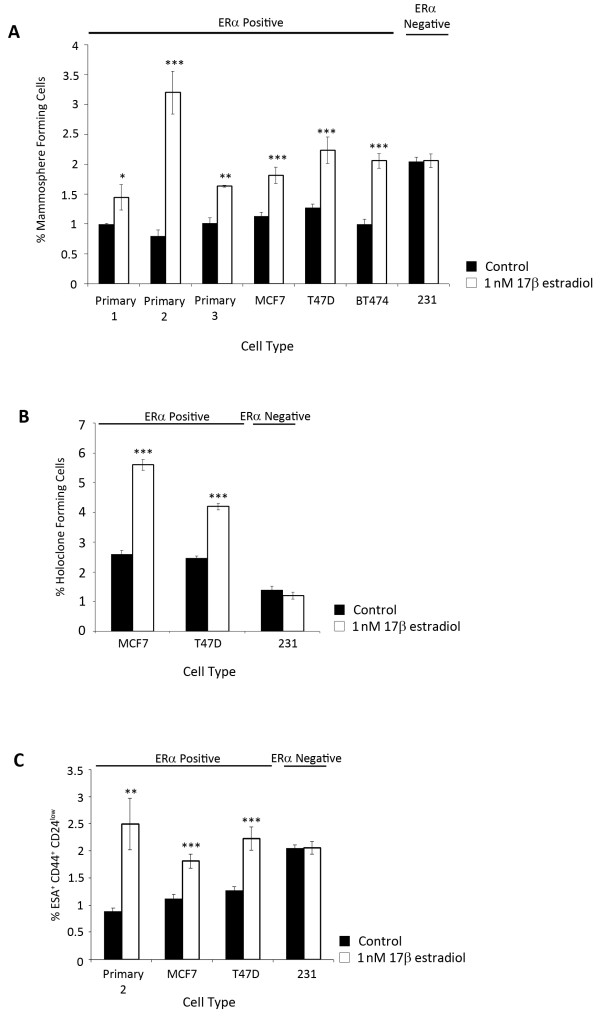
**Oestrogenic effects on cancer stem cells**. Cells were cultured for 48 hours in a monolayer in the presence of 1 nM 17β-estradiol or vehicle control and CSC assays were performed in ER positive cells (primary, MCF7, T47D and BT474) and ER negative cells (231). (**A**) Mammosphere formation, (**B**) holoclone formation and (**C**) expression of ESA^+ ^CD44^+ ^CD24^low ^were measured. Means plotted ± SEM, *P < 0.05, **P < 0.01, ***P < 0.001.

The effect of 17β-estradiol on the CSC proportion was assessed in three ER positive patient derived cell samples, three ER positive cell lines (MCF7, T47D and BT474) and an ER negative cell line (MDA-MB-231). Following oestrogen deprivation for six days and then 48 hours culture in 1 nM 17β-estradiol, the mammosphere forming cell (MFC) number was significantly increased in all ER positive but not ER negative primary cells and cell lines (Figure 1A). Holoclone forming cell (HFC) number also significantly increased in the ER positive but not ER negative cell lines (Figure 1B). ER positive cells also contained a significantly higher number of ESA^+^CD44^+^CD24^low ^cells following the 48 hour exposure to 17β-estradiol compared to control conditions (Figure 1C).

**Figure 2:**
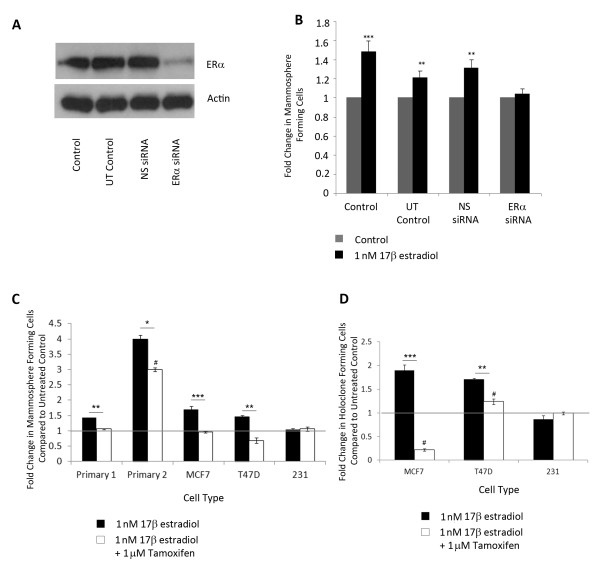
**Knock-down or inhibition of ER signalling blocks the effect of 17β-estradiol**. (**A**) Representative Western blot showing ER knock-down with siRNA. (**B**) Cells were exposed to oestrogen and the effect of ER siRNA was assessed with mammosphere culture. Cells were cultured for 48 hours in the presence of 1 nM 17β-estradiol ± tamoxifen before CSC assays were performed. (**C**) Mammosphere formation and (**D**) holoclone formation were assessed. Fold change is normalised to control, untreated cells represented as line. Means plotted ± SEM, *P < 0.05, **P < 0.01, ***P < 0.001 compared to E2 treated. # *P *< 0.05 compared to control cells.

To verify that this effect was due to activation of oestrogen signalling we first knocked down ER gene expression using siRNA (Figure 2A). In cells cultured in the presence of ER siRNA and 17β-estradiol, no significant increase in MFC is seen, establishing ER as the major player in the oestrogenic response (Figure 2B).

Next, to block ER signalling in primary cells and other cell lines, cells were cultured in the presence of oestrogen and the anti-oestrogen tamoxifen. This treatment efficiently blocked 17β-estradiol effects on ER positive patient derived cells and cell lines (Figure 2C) although primary 2 remained raised compared to control cells. HFC was also reduced in cell lines (Figure 2D) but no changes in MFC or HFC number were seen in ER negative cells.

### Breast cancer stem cells have low expression of ER

**Figure 3:**
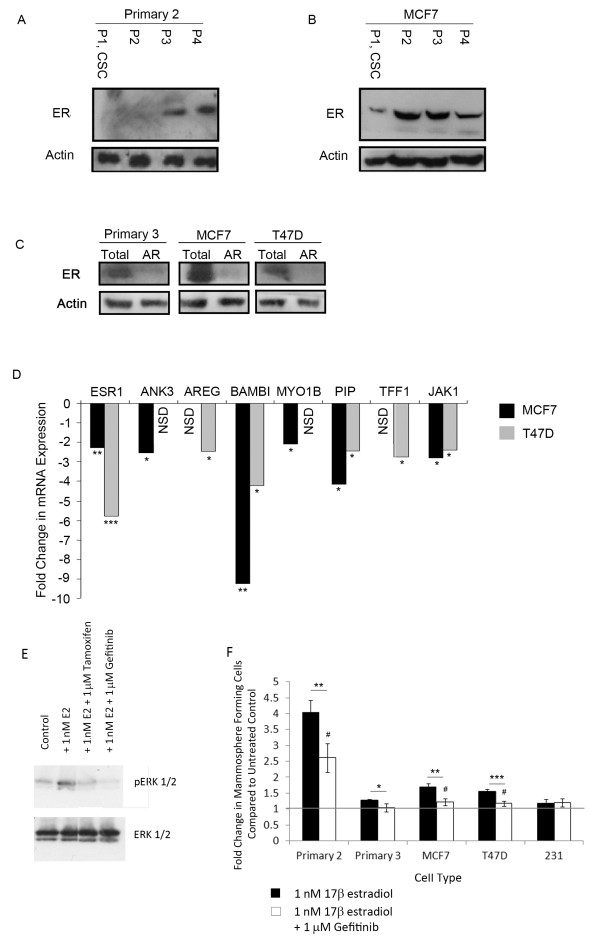
**Breast cancer stem cells have low expression of ER**. Cells were sorted based on surface markers to enrich for CSC. Representative Western blot showing ER expression in sorted primary metastatic cells (**A**) and MCF7 cells (**B**). Anoikis resistant (AR) cells from primary metastatic and cell lines (MCF7 and T47D) were collected for protein and RNA. (**C**) Representative Western blot showing expression of ER in total versus AR cells, (**D**) mRNA expression level of ER (ESR1) and responsive genes in AR cells compared to total population. (**E**) Representative Western blot of ERK and phosphorylated (actived) ERK following culture for 48 hours in monolayer ± 1 nM 17β-estradiol ± 1 μM tamoxifen or gefitinib. (**F**) Mammosphere formation was assessed following culture with 1 nM 17β-estradiol ± gefitinib. Fold change is normalised to control, untreated cells represented as line. Means plotted ± SEM, *P < 0.05, **P < 0.01, ***P < 0.001 compared to E2 treated. # *P *< 0.05 compared to control cells.

In order to demonstrate whether the effects of oestrogen and tamoxifen were direct, ER positive patient derived cells (primary 2) and MCF7 cells were FACS sorted to assess the oestrogen receptor status of the CSC enriched ESA^+^CD44^+^CD24^low ^sub-population (Additional file 3, Figure S2 and Additional file 4, Figure S3 show gating procedure and example. For more details see [18]). Cells were then lysed and ER expression was assessed by Western blot. In both cases, CSC-enriched cells (Population 1, ESA^+^44^+^24^low^) had very low or absent receptor expression compared to more differentiated tumour cells (Figure 3A, B). Populations 2 to 4 (differentiated tumour cells) expressed between 1.7- and 8-fold more ER in the primary cells and between 1.5- and 3.5-fold more ER in MCF7 cells (*P *< 0.05).

Collection of anoikis resistant (AR) cells is another method previously reported to enrich for the CSC population [18,19]. Primary cells taken from an ER positive patient derived sample (primary 3) and ER positive cell lines were plated in non-adherent culture for 16 hours before protein and RNA were prepared from the total and AR populations. In all cases the AR population showed low/no expression of ER assessed by Western blot (Figure 3C) and qRT-PCR and several fold lower levels of expression of known oestrogen responsive genes compared to total cell populations (Figure 3D). These findings indicate that paracrine signals from the ER positive cells are required to initiate increases in breast CSC number.

### EGFR signalling plays a role in the oestrogenic effect on breast cancer stem cells

In the normal breast, evidence suggests that the paracrine oestrogen signal occurs via amphiregulin (AREG) activation of the EGFR [6] and downstream phosphorylation of ERK [20]. We, therefore, measured activation of ERK signalling by ER and assessed the effect of the specific EGFR inhibitor gefitinib. Activation of ER signalling with 17β-estradiol caused increased phosphorylation of ERK which could be abrogated by treatment with tamoxifen or gefitinib, suggesting that ligand dependent ER signalling transactivates EGFR to phosphorylate ERK (Figure 3E). In mammosphere culture, gefitinib significantly reduced the effect of oestrogen in ER positive primary cells and lines (Figure 3F). However, with the exception of primary 3, the MFC remained significantly higher compared to control cultures which were not exposed to oestrogen. These findings support the hypothesis that EGFR mediated paracrine signalling plays a role in the response to oestrogen but suggests other paracrine signals are also important.

### Notch signalling is involved in the oestrogenic effect on breast cancer stem cells

**Figure 4:**
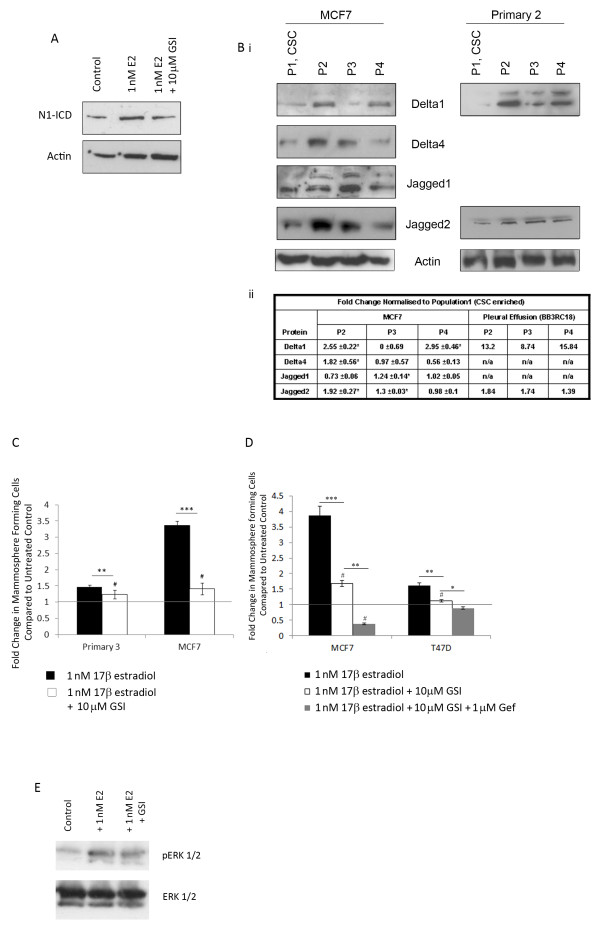
**Systemic oestrogen signalling is mediated by EGFR and Notch**. (**A**) Representative Western blot showing expression of cleaved (active) Notch1 (N1-ICD) following culture ± 1 nM 17β-estradiol ± 10 μM GSI. (**Bi**) Representative Western blot showing expression of Notch ligands in sorted MCF7 cells (left) and, where available, metastatic cells (right). (**Bii**) Densitometric analysis of three independent repeats of MCF7 sorting and of a single experiment for primary cells. Comparisons between population 1 (CSC enriched) and other populations are displayed. (**C **and **D**) Mammosphere formation was assessed following culture with 1 nM 17β-estradiol ± gamma secretase inhibitor (GSI) alone and in combination with gefitinib. Fold change is normalised to control, untreated cells represented as line. (**E**) Representative image of protein levels of ERK and phosphorylated (actived) ERK following culture for 48 hours in monolayer ± 10 μM GSI. Means plotted ± SEM, *P < 0.05, **P < 0.01, ***P < 0.001 compared to E2 treated. # *P *< 0.05 compared to control cells.

Next, we investigated Notch signalling as another likely paracrine pathway since it has been reported [21] and we confirm here that Notch1 signalling activity increases following exposure to oestrogen (Figure 4A). Ligands of the Notch pathway are expressed at significantly lower levels in the CSC enriched sub-populations (Figure 4B) similar to the EGF ligand amphiregulin (Figure 3D) suggesting signalling may be initiated by ligands derived from the non-CSC sub-population. We predicted that Notch1 receptor signalling has a role in the oestrogenic response of breast CSCs and tested this using a gamma secretase inhibitor (GSI) known to specifically target Notch1 signalling [18]. GSI significantly reduced the oestrogenic effect on MFC in primary cells and MCF7 cells (Figure 4C) but the MFC number remained significantly increased compared to control MS cultures. However, GSI used in combination with gefitinib caused MFC number to fall below that seen in control conditions (Figure 4D). GSI had no effect on ERK phosphorylation, (Figure 4E) suggesting that the Notch and EGFR signalling pathways have distinct roles in the mediation of local signalling. We conclude from these data that both EGF and Notch1 receptor signalling are paracrine mediators of the effects of systemic oestrogen on breast CSC activity.

### Oestrogen stimulation increases tumour initiating cell number

**Figure 5:**
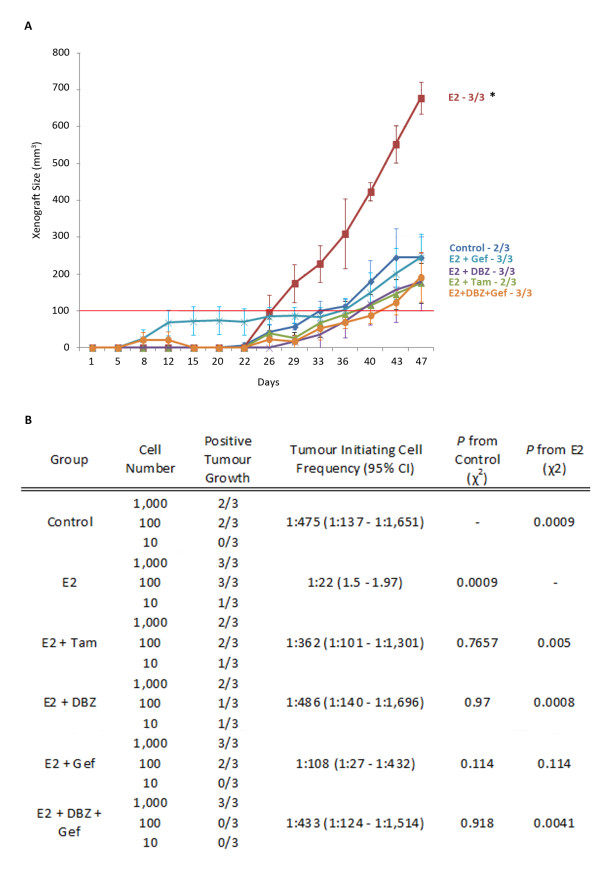
***In vivo *assessment of oestrogenic effect on breast cancer stem cells**. (**A**) Growth curves for xenografts produced from 1,000 cells pre-treated ± 17β-estradiol, ± inhibitors. *P = 0.01 (**B**) *In vivo *tumour formation in each group represented as mice positive for growth/mice tested displayed for each cell number tested. Tumour initiating cell frequency (95% CI) estimates calculated from limiting dilution analysis.

The effect of 17β-estradiol on CSC activity and frequency, and its inhibition using tamoxifen, gefitinib and GSI, was assessed with limiting dilution of cells injected into NSG mice. The human cell origin of xenografts was verified using human specific pan-cytokeratin staining (see Additional file 5, Figure S4). A total of 1,000 injected cells pre-treated with 17β-estradiol initiated tumours (> 100 mm^3^) more quickly, and these tumours grew at a significantly faster rate than those formed from control cells or those treated with inhibitors (*P *< 0.001, Figure 5A).

Using L-Calc (Stem Cell Technologies, Grenoble, France), analysis of positive tumour growth (calculated as growth ≥ 100 mm^3^) across the limiting dilution series allowed estimation of the CSC number/tumour initiating cell frequency (TIF) within each treatment (Figure 5B). TIF increased from 1:475 in control cells to 1:22 in those treated with 17β-estradiol (*P *= 0.0019). Compared to 17β-estradiol treated cells those treated with tamoxifen, GSI or gefitinib + GSI showed significantly reduce TIF and levels in these cells were not significantly different to control. The effect of 17β-estradiol does not appear to be completely blocked by gefitinib and the TIF remains higher than in control cells although not significantly (1:107, *P *= 0.118). This suggests that gefitinib alone is not able to completely block the effect of oestrogen but when used in combination with a Notch inhibitor it can increase efficiency.

## Discussion

Our data show that breast CSC activity and cell surface marker expression is increased by oestrogen exposure. This effect is observed in all ER positive patient-derived primary cells and cell lines tested and can be demonstrated both *in vitro*, using mammosphere and holoclone culture, or *in vivo*, with limiting dilution analysis. We demonstrate that the CSC-enriched populations have low/no expression of ER suggesting that the effect seen is not a direct one and we establish EGF and Notch receptor signalling pathways as important paracrine mediators of oestrogen effects on CSC activity.

The data presented here are supportive of previously published work by Fillmore *et al. *[13] but contradicts other reports showing decreased CSC activity in the presence of oestrogen [14]. We hypothesise that this is due to differences in experimental design in these reports. Simões *et al. *[14] exposed cells in non-adherent mammosphere culture to oestrogen and, therefore, affected the CSC population during anoikis resistance and MS formation. In this paper, like Fillmore *et al*., we withdrew hormones and then incubated monolayer cultures of breast cancer cells with oestrogen before measurement of the CSC population changes. This suggests that in adherent culture oestrogen affects the proliferation and/or self-renewal of CSC whilst in non-adherent culture it may affect the survival or activity of CSC in a different way.

Previously published data show that sub-populations enriched for CSC have low expression of the oestrogen receptor [13,22] but our data extend these findings to show decreased ER signalling within the CSC enriched population from metastatic ER positive patient samples.

The paracrine mediation of oestrogen signalling occurs in the normal breast through the EGFR pathway which activates ERK signalling and we demonstrate that this is also true in breast cancer. The inhibition of EGFR signalling does not, however, completely block the oestrogenic effects on CSC. An additive effect was seen when Notch inhibition with GSI was combined with gefitinib. This is supportive of recently published data which show an additive effect of EGFR/Notch inhibition on CSC activity in primary human ductal carcinoma *in situ *[23].

Notch1 was seen to increase as a result of oestrogen stimulation. In the normal breast, Notch1 is thought to play a role in the early luminal progenitor cells rather than stem cells and an analogous situation may, therefore, exist in cancers, whereby oestrogen exerts its effects on early differentiation progenitors of the CSC, rather than the CSC itself. This may suggest that early progenitor cells are directed to remain in a less differentiated state or that they de-differentiated into a more CSC-like cell type.

**Figure 6:**
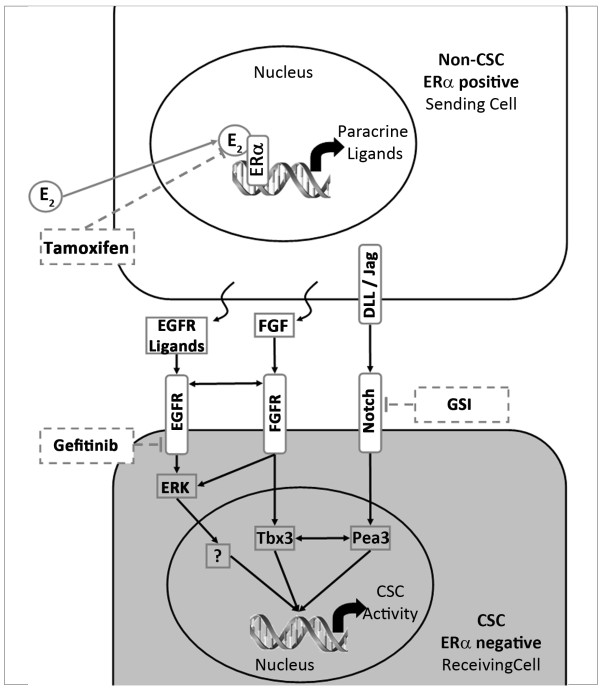
**Schematic representation of signals involved in paracrine mediation of systemic oestrogen signalling**. The signal sending cell (white) responds to oestrogen (E_2_) and initiates EGFR, FGFR and Notch ligand production. A signalling cascade is initiated within the signal receiving cell (grey) which includes, but may not be limited to ERK, Tbx3 and Pea3 signalling. This drives increased CSC activity. This signal mediation can be blocked by tamoxifen, gefitinib and gamma secretase inhibitors (GSI).

The data presented here are not directly in agreement with the previously published data showing FGF/Tbx3 signalling is responsible for this paracrine signalling in cancers [13]. There is, however, crosstalk between the two pathways and both are responsible for increased ERK signalling [20,24]. It is possible, therefore, that both pathways are involved in the oestrogenic response of the ER negative CSC/progenitor population. Furthermore, links between Tbx3 and Pea3 [25], a modulator of Notch signalling [26], support the crosstalk among EGF, FGF and Notch being responsible for CSC changes in response to oestrogen. A putative model for the paracrine signalling suggested by our results and the interactions identified by others is depicted in Figure 6.

## Conclusions

In summary, our findings provide more evidence for the ER negative/low status of breast CSC in ER positive cell lines and patient derived samples. CSC number and activity increases in response to oestrogen stimulation and, as in the normal breast, this effect is partly mediated by EGFR signalling. We have further demonstrated that Notch signalling also plays a role in the stimulation of CSC expansion by oestrogen. The paracrine signals that mediate the oestrogenic effects on CSC suggest a role for EGFR and Notch signalling in endocrine resistance and may offer suitable targets for treatment of these tumours.

## Abbreviations

ALDH1: aldehyde dehydrogenase; AR: anoikis resistant; AREG: amphiregulin; BSA: bovine serum albumin; CSC: cancer stem cell; DMEM: Dulbecco's modified Eagle's medium; EGF: epidermal growth factor; EGFR: epidermal growth factor receptor; ER: oestrogen receptor alpha; ESA: epithelial specific antigen; FGF: fibroblast growth factor; GSI: gamma secretase inhibitors; HBSS: Hank's buffered saline solution; HFC: holoclone forming cell; MFC: mammosphere forming cell; MS: mammosphere; MSC: mammary stem cell; N1-ICD: Notch1 intracellular domain; NSG mice: NOD SCID IL2gammaR knock-out mice; PBS: phosphate-buffered solution; Tam: tamoxifen; TIF: tumour initiating cell frequency

## Competing interests

The authors declare that they have no competing interests.

## Authors' contributions

HH was involved in study design, carried out the majority of the practical experimentation and drafted the manuscript. BMS managed *in vivo *experimentation and assisted in manuscript preparation. LR assisted in siRNA and Western blotting experiments. SJH identified and collected samples, provided clinical details and assisted with manuscript preparation. GL provided assistance with experimental design and manuscript preparation. RBC conceived of the study and participated in the experimental design and manuscript production. All authors have read and approved the final version of this manuscript.

## Supplementary Material

Additional file 1 **Table S1**. Primary samples used in the study. Click here for file

Additional file 2 **Figure S1: Effect of 17β-oestradiol on cancer stem cell population**. Mammosphere and holoclone formation and ESA^+^CD44^+^CD24^low ^expression with varying concentrations of 17β-oestradiol. Click here for file

Additional file 3 **Figure S2: Gating protocol for cell analysis and sorting**. Displays the gating procedure adopted. Click here for file

Additional file 4 **Figure S3: Analysis of primary cells following treatment with 17β-oestradiol**. Displays FACS analysis of cells taken from patient derived sample following treatment with 17β-oestradiol. Click here for file

Additional file 5 **Figure S4: Xenograft characterization**. Human origin of xenografts was confirmed using cytokeratin staining. Click here for file
