# Supplementary material for: Oestrogen increases the activity of oestrogen receptor negative breast cancer stem cells through paracrine EGFR and Notch signalling
Source: Breast Cancer Res. 2013 Mar 8;15(2):R21. doi: 10.1186/bcr3396 (PMC3672803; doi:10.1186/bcr3396)
Supplement: Additional file 1 — Table S1. Primary samples used in the study. [file bcr3396-S1.DOC]

| Sample # | Sample Type | Grade | ER (%) | PR (%) | Ki67 (%) | HER2 |
| --- | --- | --- | --- | --- | --- | --- |
| Primary 1 | PE | 3 | 95 | 25 | 42.3 | 1+ |
| Primary 2 | Asc | NR | 95 | 25 | 5.8 | 1+ |
| Primary 3 | PE | 2 | 90 | 98 | 19 | 1+ |

Table S1: Primary samples used in the study.

PE: Pleural effusion, Asc: Ascitic fluid, NR: data not recorded
