# Supplementary figures and images for: Oestrogen increases the activity of oestrogen receptor negative breast cancer stem cells through paracrine EGFR and Notch signalling
Source: Breast Cancer Res. 2013 Mar 8;15(2):R21. doi: 10.1186/bcr3396 (PMC3672803; doi:10.1186/bcr3396)

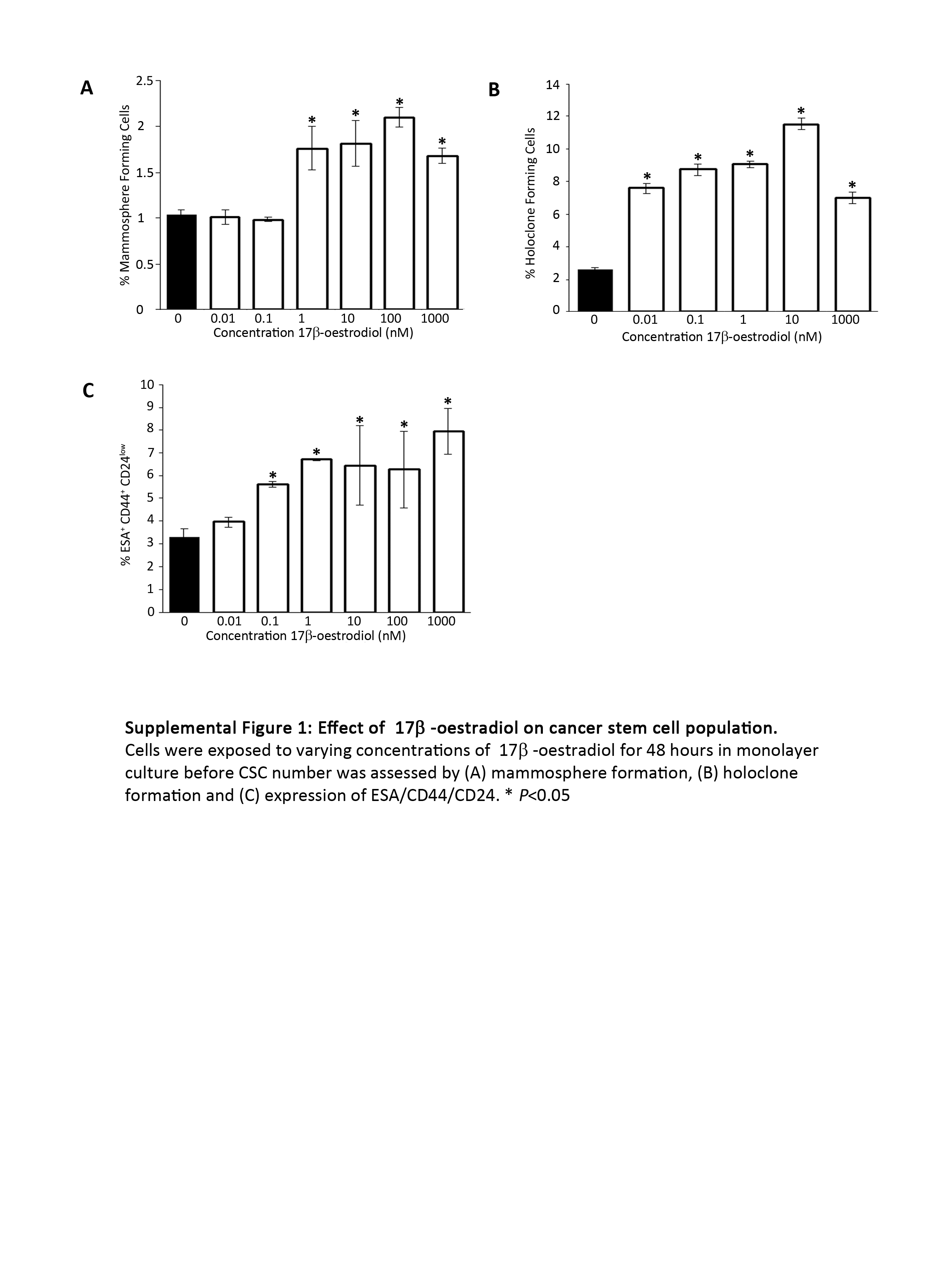

Supplement: Additional file 2 — Figure S1: Effect of 17β-oestradiol on cancer stem cell population. Mammosphere and holoclone formation and ESA+CD44+CD24low expression with varying concentrations of 17β-oestradiol. [file bcr3396-S2.TIFF]

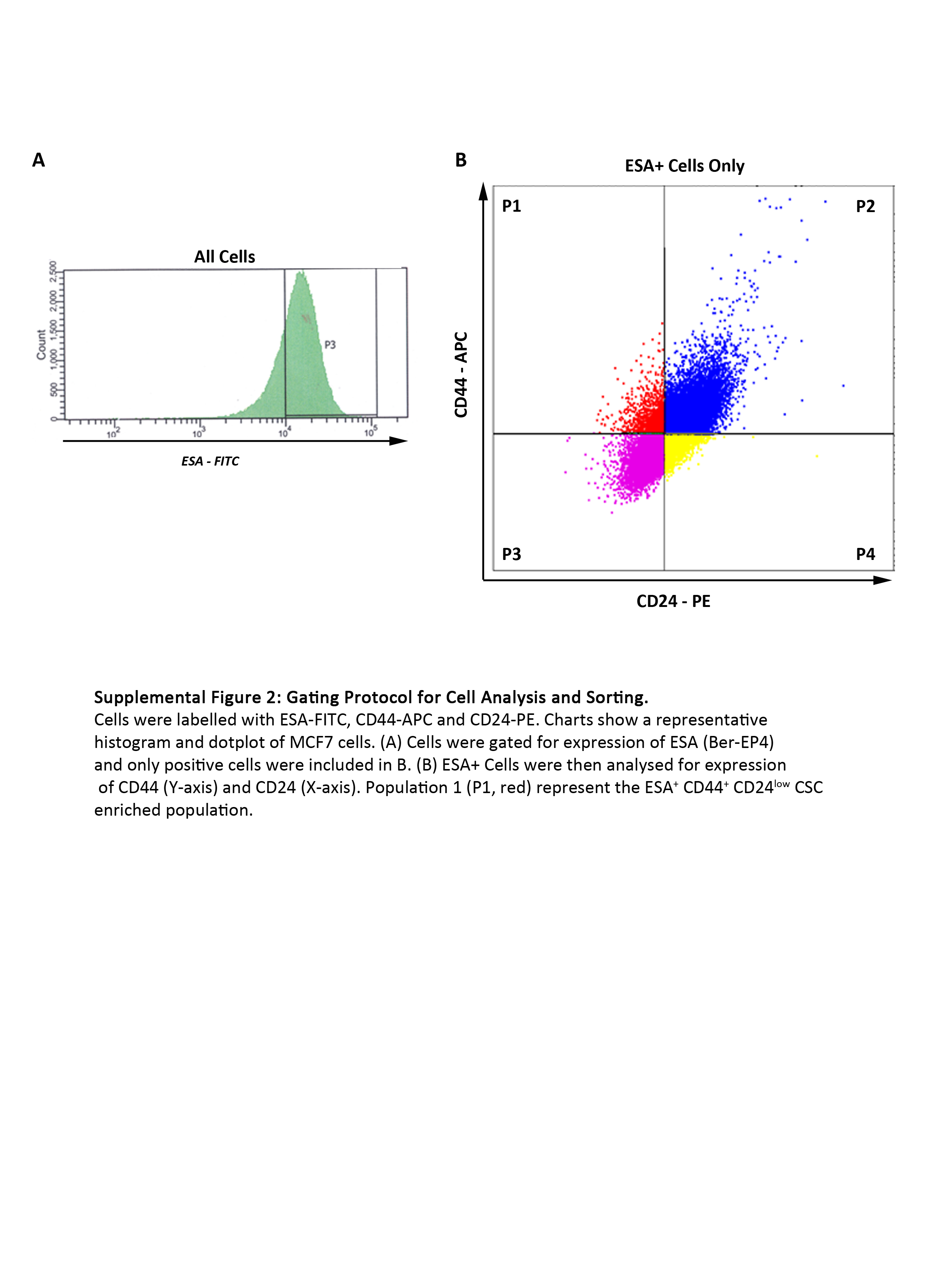

Supplement: Additional file 3 — Figure S2: Gating protocol for cell analysis and sorting. Displays the gating procedure adopted. [file bcr3396-S3.TIFF]

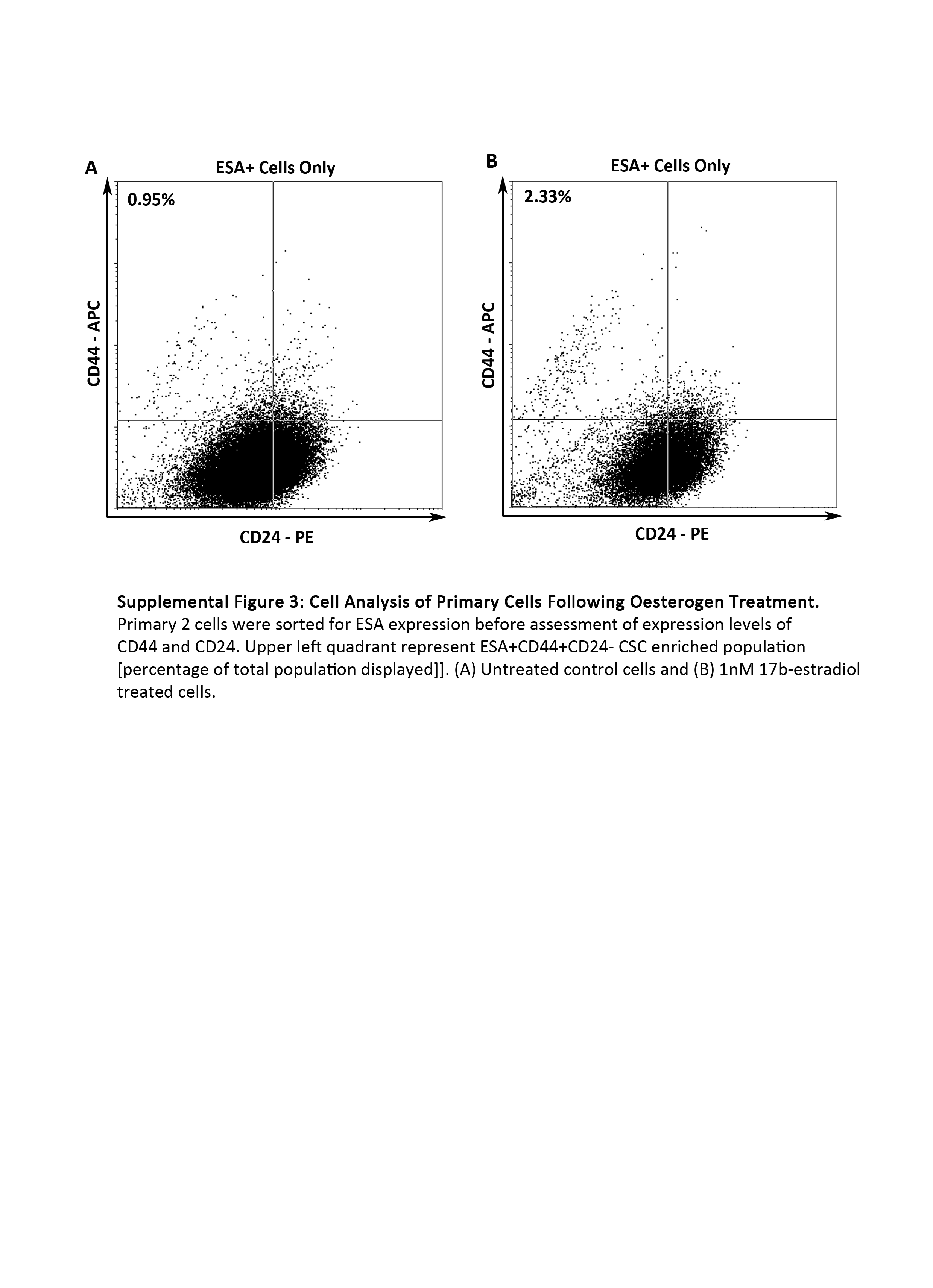

Supplement: Additional file 4 — Figure S3: Analysis of primary cells following treatment with 17β-oestradiol. Displays FACS analysis of cells taken from patient derived sample following treatment with 17β-oestradiol. [file bcr3396-S4.TIFF]

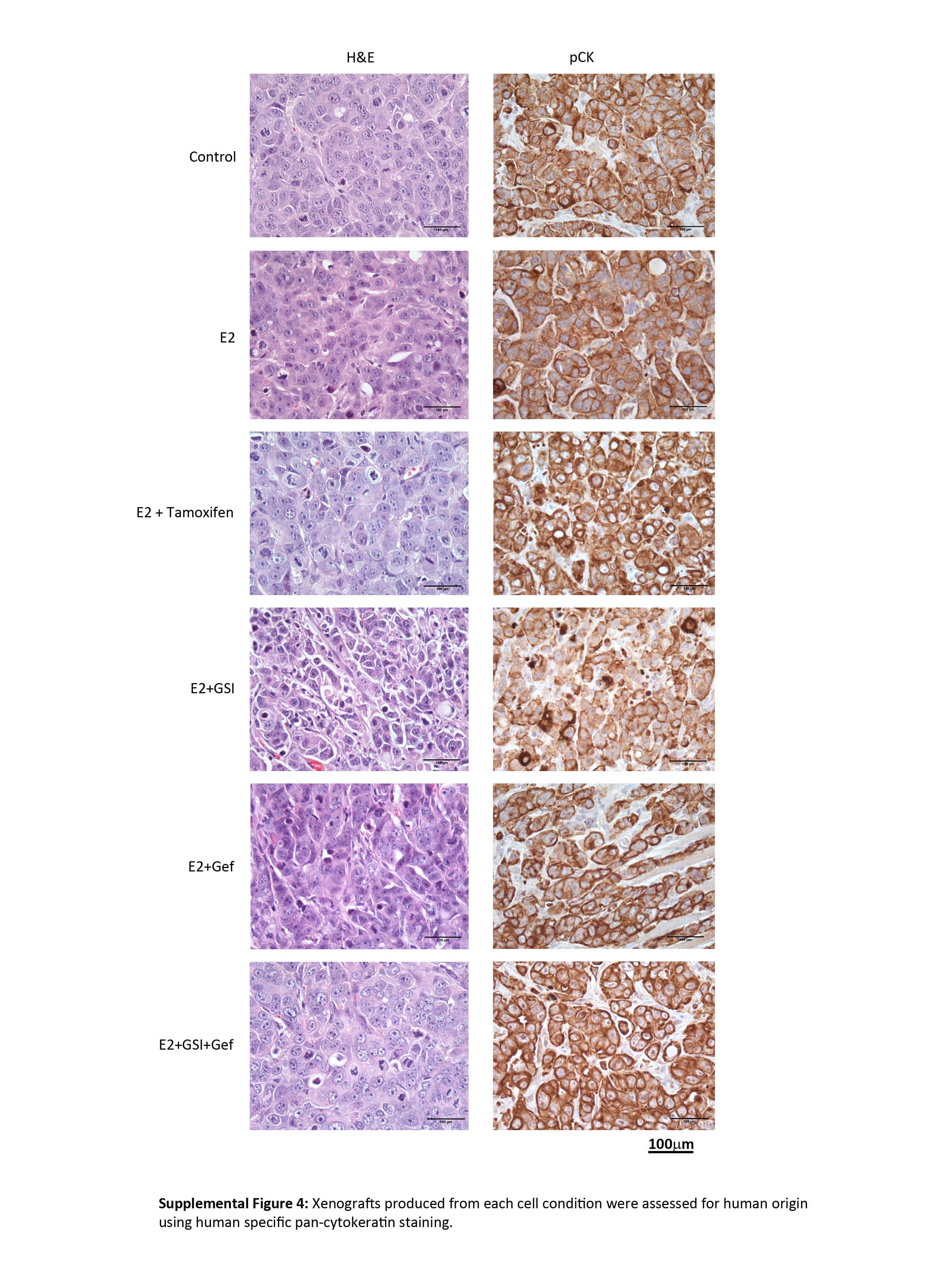

Supplement: Additional file 5 — Figure S4: Xenograft characterization. Human origin of xenografts was confirmed using cytokeratin staining. [file bcr3396-S5.TIFF]
